# Foreign body-type giant cell reaction with extensive granulation tissue and intense inflammation after chemotherapy mimicking residual lymphoma on FDG PET

**DOI:** 10.1186/s41824-022-00137-2

**Published:** 2022-09-15

**Authors:** Sakib Kazi, Stavros Raptis, Farzad Abbaspour, Wanzhen Zeng

**Affiliations:** 1 Faculty of Medicine, University of Ottawa, Ottawa, ON Canada; 2 Department of Pathology and Laboratory Medicine, Montfort Hospital, Ottawa, ON Canada; 3 Department of Nuclear Medicine, The Ottawa Hospital, Ottawa, ON Canada; 4 Division of Nuclear Medicine, Department of Medicine, Ottawa Hospital, 1053 Carling Ave., Ottawa, ON K1Y 4E9 Canada

**Keywords:** Foreign body-type giant cell reaction, [^18^F]FDG PET, Diffuse large B cell lymphoma

## Abstract

Foreign body-type giant cell reaction is typically a biological and immunological reaction to the presence of foreign bodies such as catheters, parasites or biomaterials with a collection of fused macrophages (giant cell). We reported an unusual case of [^18^F]FDG PET findings in diffuse large B cell lymphoma in the urinary bladder following incomplete resection and chemotherapy. As the restaging [^18^F]FDG PET showed intense [^18^F]FDG uptake in the urinary bladder at the resection site concerning for recurrence, the lesion was subsequently resected and histopathology showed extensive granulation tissue with foreign body-type giant cell reaction with no suspected foreign bodies or neoplasia.

## Introduction

Macrophages are myeloid immune cells that ingest and degrade dead cells and foreign materials in addition to orchestrating inflammatory processes. It is known that macrophages and their fused morphologic variants, the multinucleated giant cells which include the foreign body-type giant cells, are the dominant early responders to biomaterial implantation (Brodbeck and Anderson 2009; Sheikh et al. 2015). Biomaterial and foreign body elicit tissue and cellular responses with associated increased [^18^F]fluorodeoxyglucose (FDG) uptake have been reported (Erdoğan et al. 2013; Dong et al. 2016; Miyake et al. 2010; Rahier and Deprez 2018). Foreign body-type giant cell reaction could also be seen in rapidly dying cells with persistent activation of macrophages, with fusion and the formation of a giant cell, which has not been adequately reported in the literature (Sheikh et al. 2015; Kahn et al. 2019).

Lymphoma is routinely assessed by [^18^F]FDG positron emission tomography (PET/CT) for initial staging and following the therapy with Lugano classification. The main limitation of Lugano criteria with a high false-positive rate of FDG avid lesions is well recognized (Al Tabaa et al. 2021; Younes et al. 2017), with a false-positive proportion of 42.9% from meta-analysis (Adams and Kwee 2019). Although current NCCN guidelines indicate treatment intensification without a necessary confirmatory biopsy with elevated Deauville score, it is important to identify non-neoplastic conditions of increased [^18^F]FDG avidity, including chemotherapy-induced inflammatory process due to extensive tumor necrosis.

We present a case of intense [^18^F]FDG uptake in the urinary bladder lesion in a patient with diffuse large B cell lymphoma (DLBCL) post-initial resection and chemotherapy, with histopathologic findings of foreign body-type giant cell reaction.

## Case report

A 75-year-old male was diagnosed with stage IIIA diffuse large B cell lymphoma involving the bladder, spleen and lymph nodes. He went on to have resection of the bladder lesion in December 2020. The resection was incomplete as it was impossible to do a full thickness resection secondary to the involvement of the urinary bladder. Subsequently, the patient completed 6 courses of RCHOP (Rituximab, Cyclophosphamide, Doxorubicin Hydrochloride, Vincristine Sulfate, Prednisone) chemotherapy in April 2021.

**Fig. 1:**
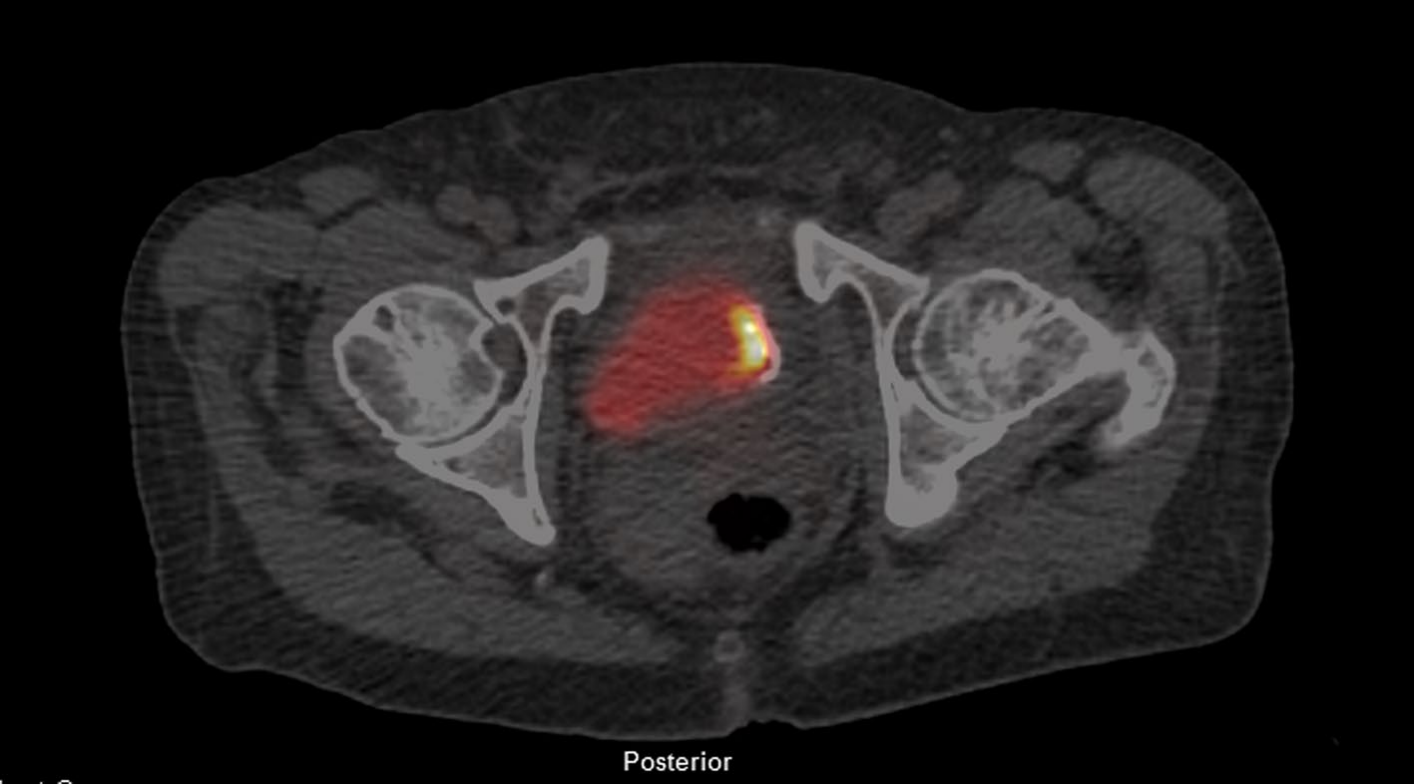
Fused transaxial images of FDG PET/CT with intense FDG uptake in the left lateral urinary bladder wall (SUV_max_ = 67.9) and calcification, at the prior resected and treated DLBCL

**Fig. 2:**
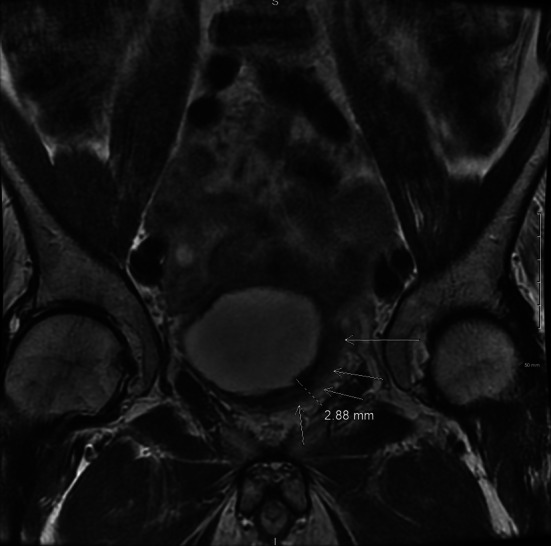
T2-weighted MRI images showed nonspecific irregular thickening of the left lateral bladder wall (arrows)

**Fig. 3:**
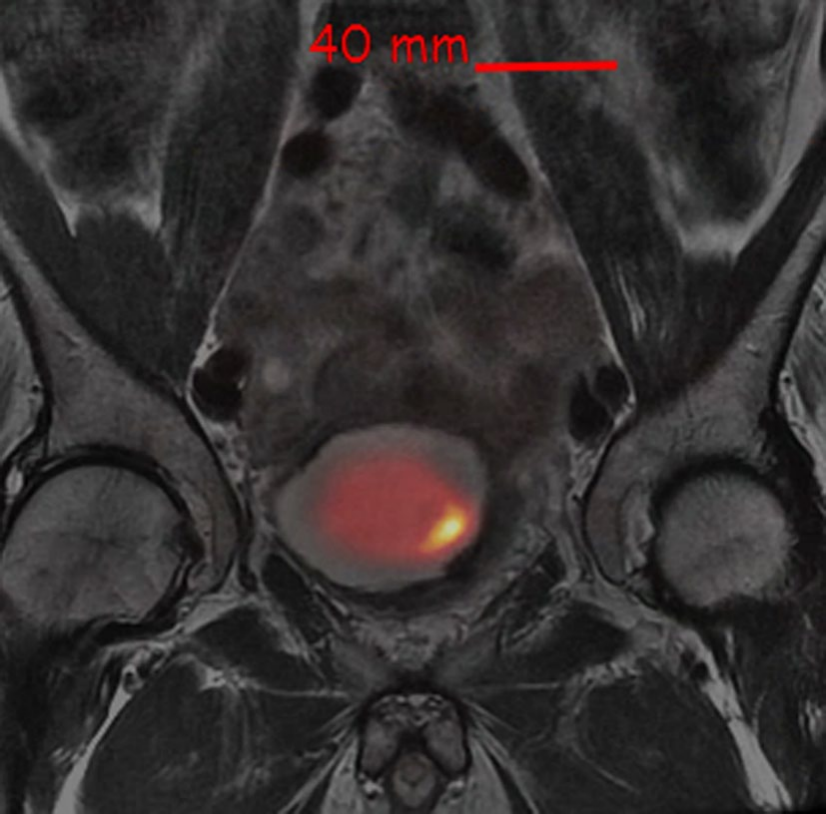
Fused PET and MRI, using bone as the landmark

On post-treatment [^18^F]FDG PET/CT in May 2021, an intensely hypermetabolic bladder wall lesion at the prior resection site was identified with a maximum standard uptake value (SUV_max_) of 67.9 (SUV_max_ of radiourine was 32.3), with a ratio of 30.9 and 25.1 when scaled to uptake of blood pool and liver (Fig. 1). A follow-up magnetic resonance imaging (MRI) was performed which showed irregular, non-masslike T2 hypointensity thickening of the left lateral bladder wall extending to the trigone (Fig. 2), corresponding to the hypermetabolic lesion on [^18^F]FDG PET (Fig. 3). Both PET and MRI were unable to definitively diagnose or exclude residual/recurrent DLBCL.

**Fig. 4:**
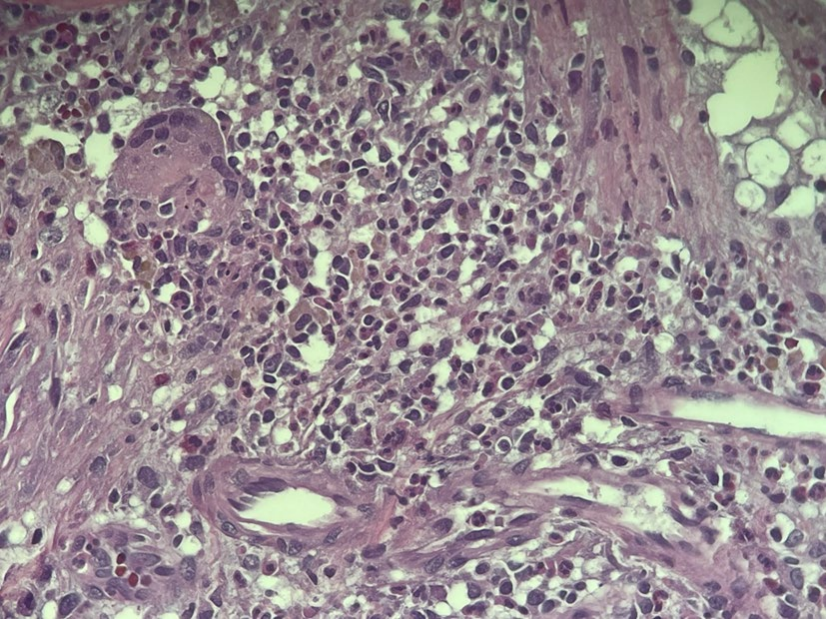
Hematoxylin and eosin stain at 400× magnification with extensive vascularization and foreign body-type giant cell reaction

The patient was brought back to the operating room for a repeat transurethral resection of the suspected bladder lesion. On pathology, extensive granulation tissue with foreign body-type giant cell reaction as well as acute and chronic inflammation was identified (Fig. 4). There was absence of neoplasia, and the patient was asymptomatic and disease free on the following-up one year post-[^18^F]FDG PET.

## Discussion

We reported a case of foreign body-type giant cell reaction with intense [^18^F]FDG uptake following chemotherapy in a patient with DLBCL, without the presence of a foreign body. The finding is thought to be due to rapid, extensive tumor necrosis in response to chemotherapy, with persistent accumulation and activation of macrophages to clean up the dying cells, as evidenced by the presence of extensive vascularization on histological examination. The “foreign body” in our case was the actual tumor dying rapidly**.** Typically, giant cells are formed by fusion of various cells such as macrophages, epithelioid cells, histiocytes and monocytes to become multinucleate cells that surround the foreign material or inflamed tissue (Brodbeck and Anderson 2009).

Although foreign body-type giant cell reaction with biological and immunological response to foreign bodies has been more frequently reported (Brodbeck and Anderson 2009; Sheikh et al. 2015), it has also been reported in the setting of tumor necrosis, probably mediated by cytokines such as tumor necrosis factor (Brooks et al. 2019). In a patient with renal cell carcinoma following cryoablation, Kahn et al. (2019) described a surgically resected retroperitoneal perinephric mass thought to be recurrent renal cell carcinoma that was pathologically confirmed as tumefactive fat necrosis with multinucleate giant cell reaction. In a historical study assessing changes of metastatic lymph nodes of the squamous cell type after radiation (McGregor 1934), Dr. McGregor stated “Giant cells of the foreign body type were always found in radiated and non-radiated nodes wherever necrotic cancer came directly in contact with lymph node tissues” and “The presence of these giant cells must be regarded as a favorable sign.” In a study of 5 patients with lymphoma, development of post-chemotherapy histiocyte-rich pseudotumor was reported (Goebel et al. 2021). It was thought that in a setting of massive tumor cell apoptosis following chemotherapy, the normal clearance mechanisms are overwhelmed and proinflammatory intracellular contents, known as damage-associated molecular patterns, are released from the cell, inducing histiocyte recruitment.

The inflammatory process with foreign body-type giant cell reaction is known to be associated with increased [^18^F]FDG uptake on [^18^F]FDG PET scan (Erdoğan et al. 2013; Dong et al. 2016; Miyake et al. 2010; Rahier and Deprez 2018), with reported SUV_max_ ranged at 9.1–28.0 (average: 17.6). In the present case, the markedly intense uptake (SUV_max_ of 68) is rarely observed, likely indicative of rapid tumor response to chemotherapy with extensive tumor necrosis and active inflammatory process.

In conclusion, highly [^18^F]FDG avid lesion with foreign body-type giant cell reaction could occur without the presence of a foreign body and may be indicative of rapidly dying tumor cells in initial response to chemotherapy. We concluded that the intensity of [^18^F]FDG uptake in the suspected lesion in diffuse large B cell lymphoma following chemotherapy may be nonspecific and should not be used as the diagnostic criterion for malignancy.
